# P-1511. *In Vitro* Activity of Gepotidacin Against Molecularly Characterized *Neisseria gonorrhoeae* Isolates Exhibiting Reduced Susceptibility to Antibacterial Agents, 2018-2021

**DOI:** 10.1093/ofid/ofae631.1680

**Published:** 2025-01-29

**Authors:** Mark Estabrook, Renuka Kapoor, Didem Torumkuney, Henry Li, Daniel F Sahm

**Affiliations:** IHMA, Schaumburg, Illinois; GSK, Atlanta, Georgia; GSK, Atlanta, Georgia; IHMA, Schaumburg, Illinois; IHMA, Schaumburg, Illinois

## Abstract

**Background:**

Gepotidacin (GEP) is a novel, bactericidal, first-in-class triazaacenaphthylene antibacterial that inhibits bacterial DNA replication by a unique mechanism of action, distinct binding site, and provides a well-balanced inhibition (for most uncomplicated urinary tract infection (uUTI) uropathogens and *N. gonorrhoeae*)) of two different Type II topoisomerase enzymes. We present the activity of GEP against molecularly characterized *N. gonorrhoeae* isolates collected from patients in Australia, India, and the United States from 2018-2021 as part of a GEP global gonococcal surveillance study.

**Figure:**
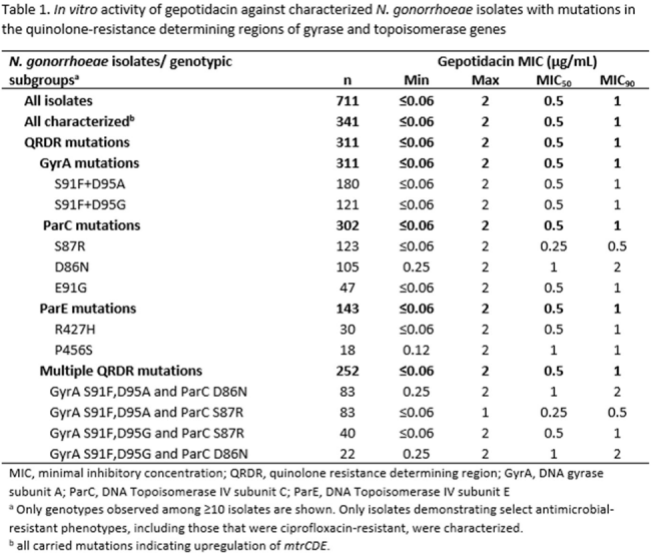

**Methods:**

*N. gonorrhoeae* isolates were tested for susceptibility to GEP and comparator agents by CLSI agar dilution method and analyzed using CLSI 2024 breakpoints. Isolates that met predefined selection criteria, which included GEP MIC values >1 µg/mL or nonsusceptible or resistant (R) to select comparators, were characterized by whole genome sequencing.

**Results:**

A total 711 *N. gonorrhoeae* isolates were collected and of these 341 (48%) were characterized. The GEP MICs for both populations of isolates ranged from ≤0.06 to 2 µg/mL; MIC_90_ of 1 µg/mL (Table 1). Of 324 ciprofloxacin-R isolates, 105 (32.4%) had a mutation of D86N in ParC; this mutation was associated with a GEP MIC_90_ of 2 µg/mL (range= 0.25-2 µg/mL). Isolates with this mutation also carried mutations in the quinolone-resistance determining region (QRDR) of GyrA as well as mutation(s) suggesting the upregulation of the MtrCDE efflux pump. When categorizing isolates by QRDR mutation, ParC D86N was the only mutation associated with a GEP MIC_90_ >1 µg/mL. A myriad of resistance mechanisms relevant to penicillin, azithromycin, and tetracycline, were identified among the characterized isolates, against which GEP MIC_90_s ranged from 1-2 µg/mL (Table 2).

**Conclusion:**

GEP demonstrated potent *in vitro* activity against *N. gonorrhoeae* isolates, including those that were not susceptible to comparator agents. GEP’s MIC_90_ value relative to all isolates, and those with other QRDR mutations in this study, was one dilution higher (2 versus 1 µg/mL) against isolates that carried ParC D86N, which is known to be important for GEP binding.

**Disclosures:**

**Mark Estabrook, MS**, Pfizer, Inc.: Advisor/Consultant **Renuka Kapoor, PhD**, GSK: Employee|GSK: Stocks/Bonds (Public Company) **Didem Torumkuney, PhD**, GSK: Employee|GSK: Stocks/Bonds (Public Company) **Henry Li, MS in Biotechnology**, Pfizer, Inc.: Advisor/Consultant **Daniel F. Sahm, PhD**, Pfizer, Inc.: Advisor/Consultant

